# Supplementary material for: Regulation of plant phototropic growth by NPH3/RPT2-like substrate phosphorylation and 14-3-3 binding
Source: Nat Commun. 2021 Oct 21;12:6129. doi: 10.1038/s41467-021-26333-5 (PMC8531357; doi:10.1038/s41467-021-26333-5)
Supplement: Supplementary file 1 — Supplementary Information [file 41467_2021_26333_MOESM1_ESM.pdf]

SUPPLEMENTARY INFORMATION

Regulation of Plant Phototropic Growth by NPH3/RPT2-like Substrate Phosphorylation and 14-3-3 Binding

Stuart Sullivan, Thomas Waksman, Dimitra Paliogianni, Louise Henderson, Melanie Lütkemeyer, Noriyuki Suetsugu and John M. Christie

Supplementary Figures 1-7

Supplementary Table 1

Supplementary Figures

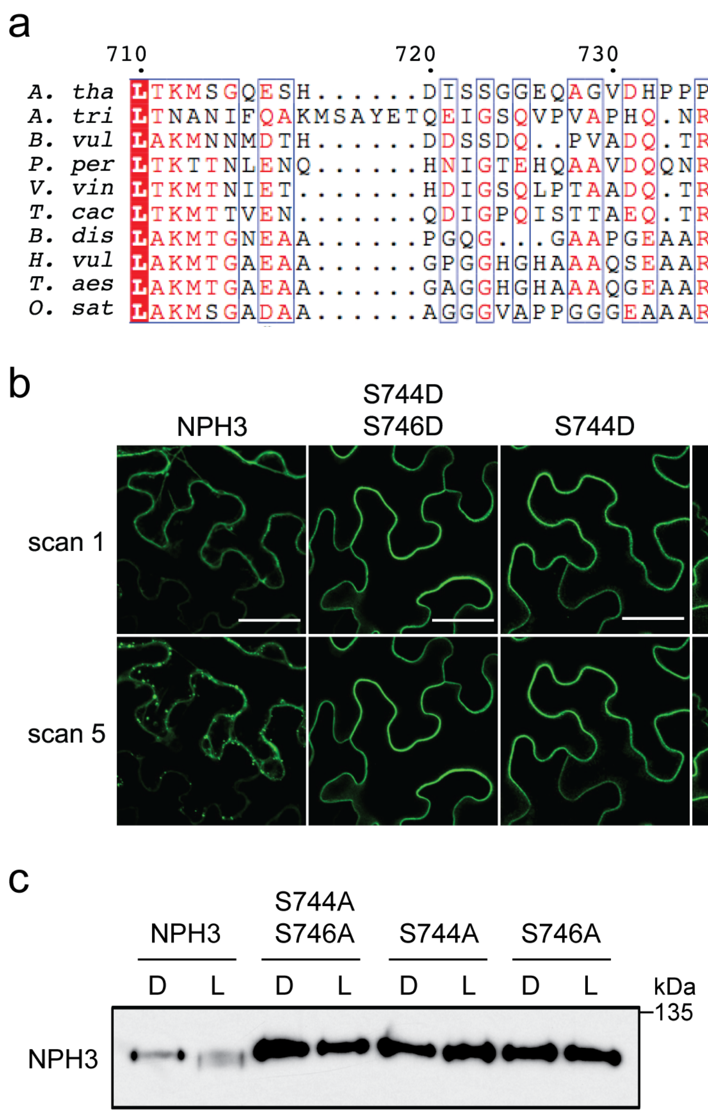

Supplementary Figure 1. Mutational analysis of NPH3 phosphorylation sites. **a** Amino acid alignment of the C-terminus of NPH3 from *Arabidopsis thaliana (A. tha), Amborella trichopoda (A. tri), Beta vulgaris (B. vul), Prunus persica (P. per), Vitis vinifera (V. vin), Theobroma cacao (T. cac), Brachypodium distachyon (B. dis), Hordeum vulgare (H. vul) Triticum aestivum (T. aes) and Oryza sativa (O. sat)*. The two conserved serine residues (*A. tha*, S744 and S746) are indicated by arrowheads. **b** Confocal images of GFP-NPH3 (NPH3) and phosphorylation site mutants S744D S746D, S744D and S746D transiently expressed in leaves of *N. benthamiana*. Plants were dark-adapted before confocal observation and images acquired immediately (scan 1) and after repeat scanning with the 488 nm laser (scan 5). Bar, 50 µm. **c** Immunoblot analysis of protein extracts from leaves of *N. benthamiana* transiently expressing GFP-NPH3 (NPH3) and phosphorylation site mutants S744D S746D, S744D and S746D. Plants were dark-adapted and maintained in darkness (D) or irradiated with 20 µmol m^{-2} s^{-1} of blue light for 15 min (L). Protein extracts were probed with anti-GFP antibodies. Experiments were repeated at least twice with similar results.

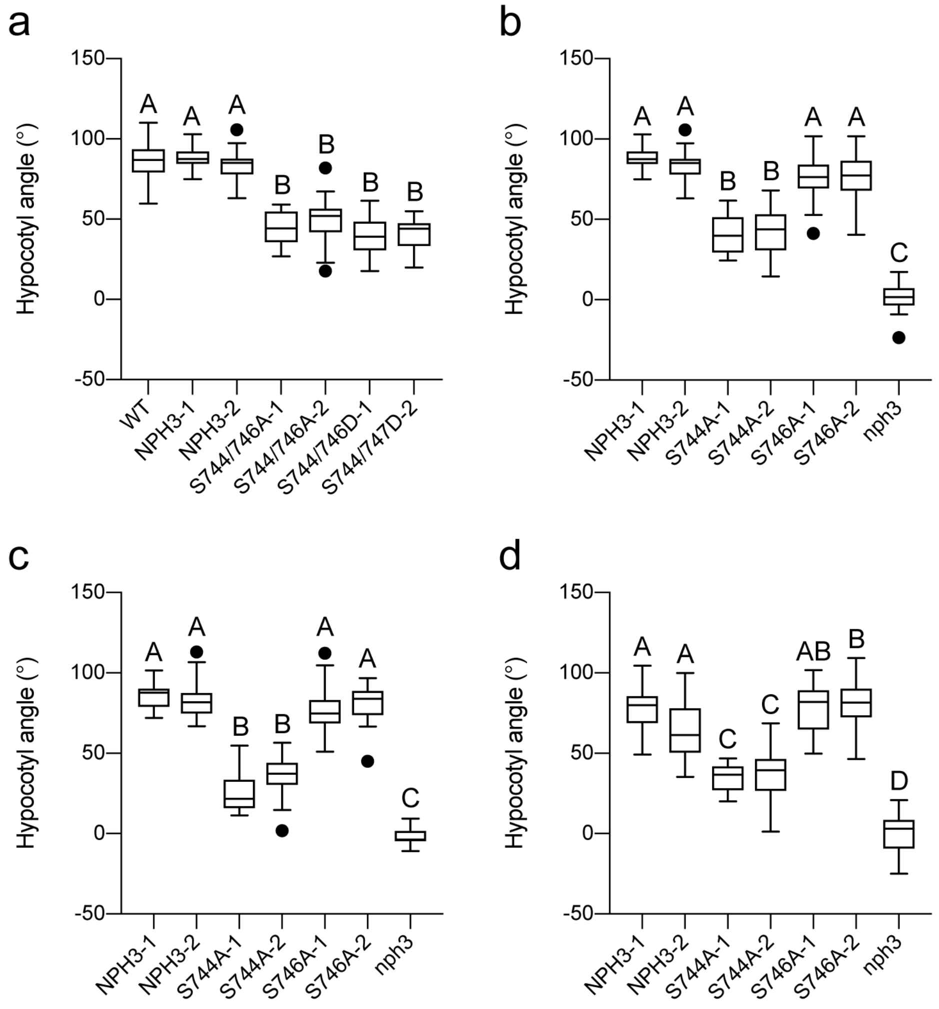

Supplementary Figure 2. Phot1 phosphorylation of NPH3 promotes functionality. Box-and-whisker plots showing the final angle of hypocotyl curvature after 240 min of irradiation. **a** Data taken from Figure 6a of seedlings irradiated with 0.5 µmol m^{-2} s^{-1} unilateral blue light. **b** Data taken from Figure 6b of seedlings irradiated with 0.5 µmol m^{-2} s^{-1} unilateral blue light. **c** Data taken from Figure 6c of seedlings irradiated with 0.05 µmol m^{-2} s^{-1} unilateral blue light. **d** Data taken from Figure 6d of seedlings irradiated with 20 µmol m^{-2} s^{-1} unilateral blue light. For the box plots, the centre line indicates the median, the bounds of the boxes indicate the 25th and 75th percentiles, whiskers extend 1.5 times the interquartile range and outliers are represented by dots. Different letters denote significant differences ($P < 0.01$, one-way ANOVA with Tukey post-test). Exact P-values are provided in the Source data file.

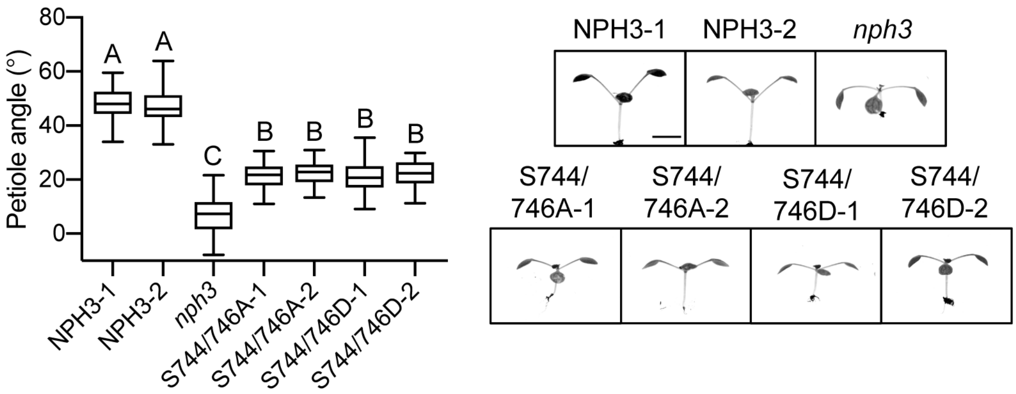

Supplementary Figure 3. Phot1 phosphorylation of NPH3 promotes functionality. Petiole positioning of *nph3* mutant and *nph3* seedlings expressing GFP-NPH3 (NPH3) or phosphorylation site mutants S744A S746A and S744D S746D. Plants were grown under 80 µmol m^{-2} s^{-1} white light for 9 d before transfer to 10 µmol m^{-2} s^{-1} white light for 5 d. Box-and-whisker plots show petiole angle from the horizontal measured for the first true leaves. The centre line indicates the median, the bounds of the boxes indicate the 25th and 75th percentiles, whiskers extend 1.5 times the interquartile range and outliers are represented by dots (n = 60 seedlings, from three independent biological replicates). Different letters denote significant differences (P < 0.01, one-way ANOVA with Tukey post-test). Exact P-values are provided in the Source data file. Representative images for each genotype are shown on the right. Bar, 5 mm.

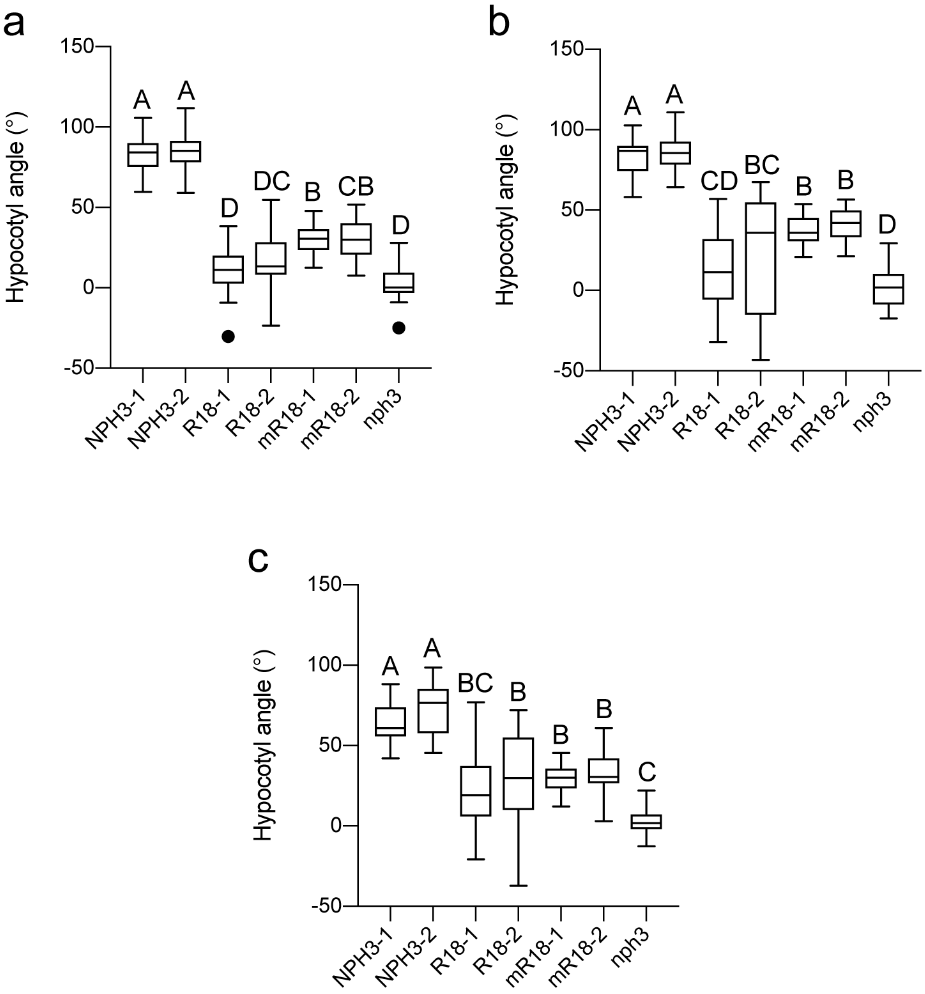

Supplementary Figure 4. Analysis of a constitutive 14-3-3 binding NPH3 variant. Box-and-whisker plots showing the final angle of hypocotyl curvature after 240 min of irradiation. **a** Data taken from Figure 7f of seedlings irradiated with 0.05 µmol m^{-2} s^{-1} unilateral blue light. **b** Data taken from Figure 7g of seedlings irradiated with 0.5 µmol m^{-2} s^{-1} unilateral blue light. **c** Data taken from Figure 7h of seedlings irradiated with 20 µmol m^{-2} s^{-1} unilateral blue light. For the box plots, the centre line indicates the median, the bounds of the boxes indicate the 25th and 75th percentiles, whiskers extend 1.5 times the interquartile range and outliers are represented by dots. Different letters denote significant differences ($P < 0.01$, one-way ANOVA with Tukey post-test). Exact P-values are provided in the Source data file.

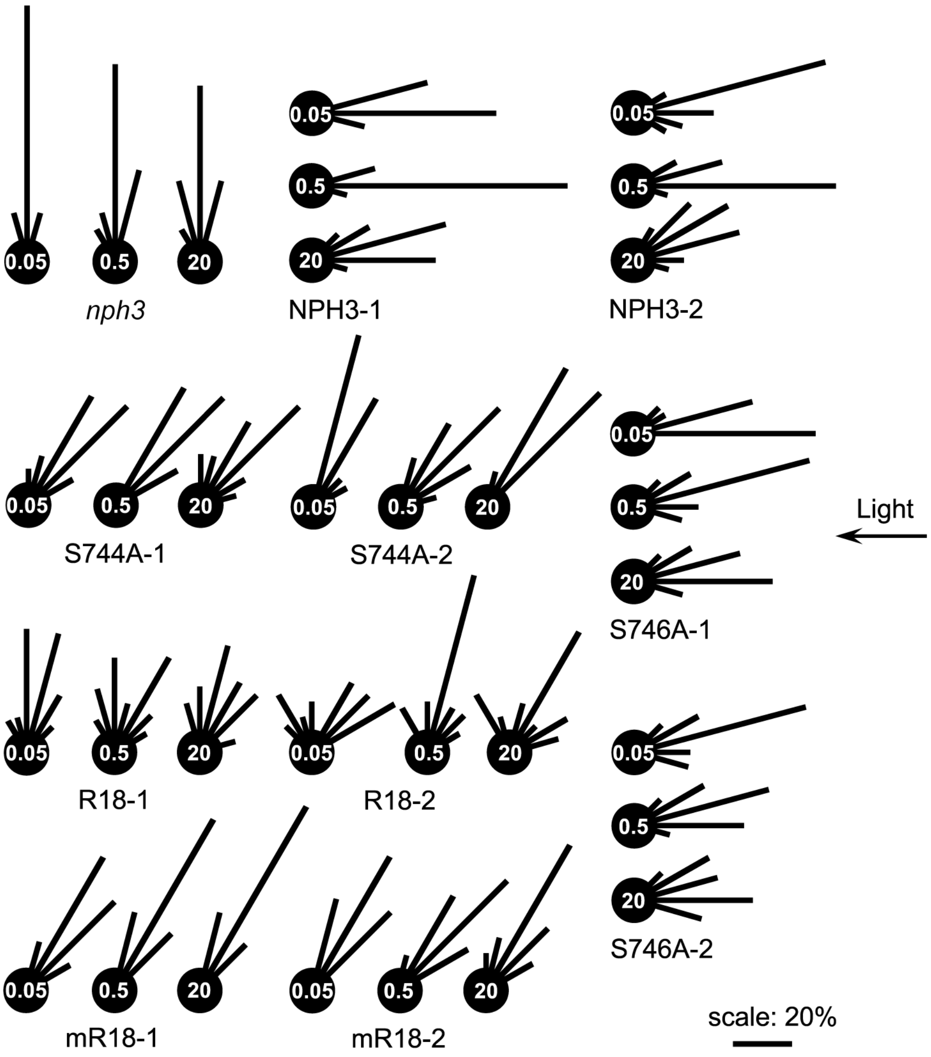

Supplementary Figure 5. Analysis of a constitutive 14-3-3 binding NPH3 variant. Circular histograms depicting hypocotyl orientation after 240 min of irradiation with 0.05 µmol m^{-2} s^{-1}, 0.5 µmol m^{-2} s^{-1} or 20 µmol m^{-2} s^{-1} unilateral blue light. Angles were grouped into 15° classes and expressed as percentages of the number of seedlings.

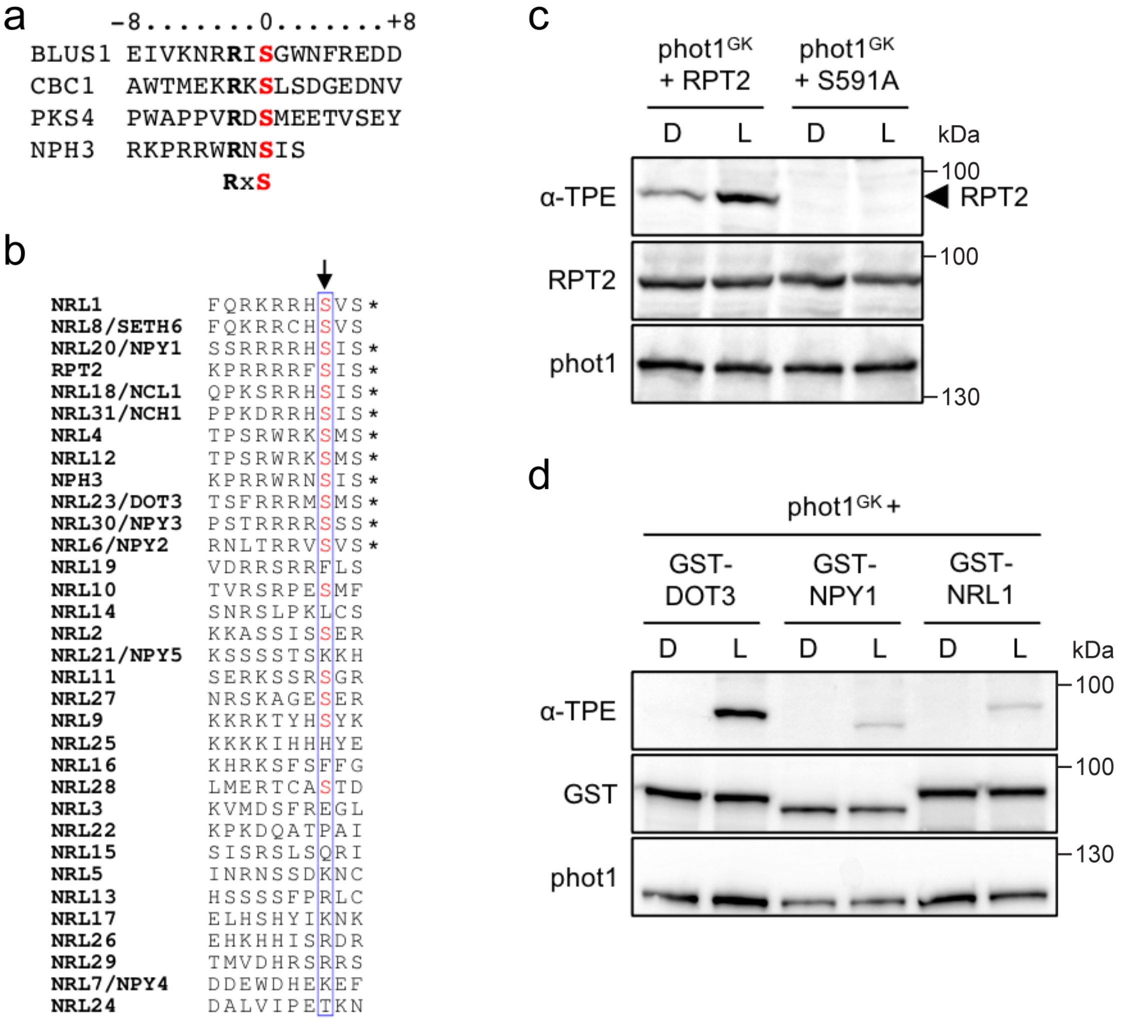

Supplementary Figure 6. Conservation of the phot1 phosphorylation sequence of NPH3**. a** Amino acid sequence alignment of the phototropin 1 substrate phosphorylation sites in BLUS1 (Takemiya et al., 2013), CBC1 (Hiyama et al., 2017), PKS4 (Schumacher et al., 2018) and NPH3. The amino acid residues are numbered relative to the phosphorylated serine residue and the PKA-like phosphorylation motif is indicated below. **b** Amino acid alignment of the last 10 residues of the *Arabidopsis* NRL protein family. The position of S744 is indicated by an arrow and sequences containing a RxS phosphorylation motif denoted with an asterisk. **c** Thiophosphorylation analysis of *in vitro* kinase assays containing gatekeeper engineered phot1 ($phot1^{GK}$) and RPT2 or RPT2-S591A. Reactions were performed in the absence (D) or presence of 20 s of white light (L), and thiophosphorylation was detected using anti-thiophosphoester antibody (α-TPE). Blots were probed with anti-GST antibody to detect GST-RPT2 and $phot1^{GK}$-GST. **d** Thiophosphorylation analysis of *in vitro* kinase assays containing $phot1^{GK}$ and GST-DOT3, GST-NPY1 OR GST-NRL1. Reactions were performed in the absence (D) or presence of 20 s of white light (L), and thiophosphorylation was detected using α-TPE. Blots were probed with anti-GST antibody to detect NRL proteins and anti-HA to detected HA-$phot1^{GK}$. The experiments in (**c**) and (**d**) were preformed twice with similar results.

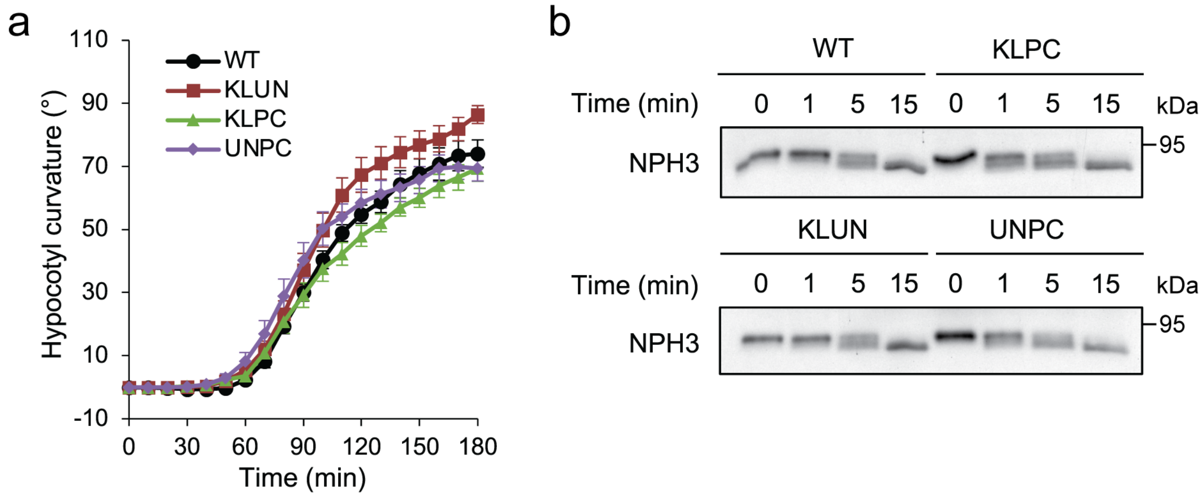

Supplementary Figure 7. Analysis of quadruple 14-3-3 mutants. **a** Phototropism of etiolated wild-type (WT) seedlings or *kappa lambda phi chi* (KLPC), *kappa lambda upsilon nu* (KLUN) and *upsilon nu phi chi* (UNPC) quadruple mutant seedlings irradiated with 0.5 μmol m^{-2} s^{-1} unilateral blue light. Hypocotyl curvatures were measured every 10 min for 3 h, and each value is the mean ± SE of 10 independent seedlings from two biological replicates. **b** Immunoblot analysis of total protein extracts from etiolated WT or KLPC, KLPC and UNPC quadruple mutant seedlings irradiated with 15 μmol m^{-2} s^{-1} of blue light for the time indicated. Blots were probed with anti-NPH3 antibodies. This experiment was repeated twice with similar results.

Primer Name	Primer Sequence (5' to 3')	Description
NPH3_S744A-F	CAAGACGATGGAGGAACGCAATTTCATGAGGATCC	Site directed mutagenesis of NPH3 serine 744 to alanine
NPH3_S744A-R	GGATCCTCATGAAATTGCGTTCCTCCATCGTCTTG	
NPH3_S746A-F	GATGGAGGAACTCAATTGCATGAGGATCCTGGGC	Site directed mutagenesis of NPH3 serine 746 to alanine
NPH3_S746A-R	GCCCAGGATCCTCATGCAATTGAGTTCCTCCATC	
NPH3_S744D-F	CAAGACGATGGAGGAACGATATTTCATGAGGATCCTG G	Site directed mutagenesis of NPH3 serine 744 to aspartic acid
NPH3_S744D-R	CCAGGATCCTCATGAAATATCGTTCCTCCATCGTCTT G	
NPH3_S746D-F	CAAGACGATGGAGGAACTCAATTGATTGAGGATCCTG GGCACG	Site directed mutagenesis of NPH3 serine 746 to aspartic acid
NPH3_S746D-R	CGTGCCCAGGATCCTCAATCAATTGAGTTCCTCCATC GTCTTG	
NPH3-S744A_S746A-F	AAGACGATGGAGGAACGCAATTGCATGAGGATCCTG GGC	Site directed mutagenesis of NPH3 serine 744 and 746 to alanine
NPH3-S744A_S746A-R	GCCCAGGATCCTCATGCAATTGCGTTCCTCCATCGTC TT	
NPH3-S744D_S746D-F	CAAGACGATGGAGGAACGATATTGATTGAGGATCCTG GGCACG	Site directed mutagenesis of NPH3 serine 744 and 746 to aspartic acid
NPH3-S744D_S746D-R	CGTGCCCAGGATCCTCAATCAATATCGTTCCTCCATC GTCTTG	
NPH3-MluI-F	CTCGAGAAACGCGTAGGGATG	Amplification of NPH3 with a C-terminal R18 or mR18 peptide sequence
NPH3-R18-BamHI-R	TTATGGATCCTCATGGCAAACACATGTTAGCTTCCAG GTCCAGCCATGACAGGTCCCTTGGAACGCAATGTGG GTTCCTCCATCGTCTTGGTTTCC	
NPH3-mR18-BamHI-R	TTATGGATCCTCATGGCAAACACATGTTAGCTTTCAG CTTCAGCCATGACAGGTCCCTTGGAACGCAATGTGG GTTCCTCCATCGTCTTGGTTTCC	
NPH3-pUCSP-KpnI-F	GAGATATCGGGTCCCGGTACCTTATGATGTGGGAATC TGAG	Gibson cloning primers to create NPH3-pUCSP plasmid
NPH3-pUCSP-BamHI-R	CTGTGTTCTCGTCGTGCCCAGGATCCTCATGAAATTG AGTTC	
GST-BamHI-DOT3-F	ATCGGATCTGGTTCCGCGTGGATCCATGAATTCAGTA TCAGCTGC	Gibson cloning primers to create pSP64-GST-DOT3 plasmid
DOT3-EcoRI-R	CAGCTATGACCATGATTACGAATTCTTATGACATTGAC ATTCTCCTC	
GST-BamHI-NPY1-F	ATCGGATCTGGTTCCGCGTGGATCCATGAAGTTCATG AAGCTAGGG	Gibson cloning primers to create pSP64-GST-NPY1 plasmid
NPY1-EcoRI-R	CAGCTATGACCATGATTACGAATTCTCACGATATCGA ATGTCTGC	
GST-BamHI-NRL1-F	ATCGGATCTGGTTCCGCGTGGATCCATGGGACTTGTT ACAGTCGG	Gibson cloning primers to create pSP64-GST-NRL1 plasmid
NRL1-EcoRI-R	CAGCTATGACCATGATTACGAATTCTCAAGAAACAGA GTGTCGTC	
RPT2_pSP64-GST_F	ATCGGATCTGGTTCCGCGTGGATCCATGGCAACAGA AGGAAAAAAC	Gibson cloning primers to create pSP64-GST-RPT2 plasmid
RPT2_pSP64_R	CAGCTATGACCATGATTACGAATTCTTAAGAGATTGA GAATCTTCGTC	
RPT2_S591A_pSP64_R	CAGCTATGACCATGATTACGAATTCTTAAGAGATTGC GAATCTTCGTC	
HA-NPH3_pSP64_F	AGGTGACACTATAGAATACAAGCTTAACAATGTACCC ATACGATGTTCCAGATTACGCTATGATGTGGGAATCT GAG	Gibson cloning primers to create pSP64-HA-NPH3 plasmid
NPH3_pSP64_R	CAGCTATGACCATGATTACGAATTCATGAAATTGAGTT CCTC	
NPH3_S744A_pSP64_R	CAGCTATGACCATGATTACGAATTCATGAAATTGCGTT CCTC	

GST-P1-pSP64-GB-F	GTGACACTATAGAATACAAGCTTAACAATGTCCCCTATACTAGGTTATTG	Gibson cloning primers to create pSP64-GST-phot1-HA plasmid
GST-P1-pSP64-GB-R	CTAGAGTCGACCTGCAGTTAAGCATAATCTGGAACATCGTAAGGATAAAAAACATTTGTTTGCAGATC	
phot1-pSP64-F	AGTCAGAAGCTTAACAATGGAACCAACAGAAAAACCATCGAC	Gibson cloning primers to create pSP64-phot1 or pSP64-phot1-HA plasmid
phot1-BamHI-R	TCAGTCGGATCCTTAAAAAACATTTGTTTGCAGATCTTCTAG	
phot1-pSP64-R	TCAGTCCTGCAGTTAAGCATAATCTGGAACATCGTAAGGATAAAAAACATTTGTTTGCAGATCTTCTAGCTC	
PHOT1_T740G-F	CACATATATGTCTTATAGGAGATTACTATCCAGG	Site directed mutagenesis of PHOT1 tyrosine 740 to glycine
PHOT1_T740G-R	CCTGGATAGTAATCTCCTATAAGACATATATGTG	

Supplementary Table 1. Sequences of DNA primers used for cloning and site directed mutagenesis.
