# Supplementary material for: Regulation of plant phototropic growth by NPH3/RPT2-like substrate phosphorylation and 14-3-3 binding
Source: Nat Commun. 2021 Oct 21;12:6129. doi: 10.1038/s41467-021-26333-5 (PMC8531357; doi:10.1038/s41467-021-26333-5)
Supplement: Supplementary file 3 — Description of Additional Supplementary Files [file 41467_2021_26333_MOESM3_ESM.pdf]

Description of Additional Supplementary Files

File name: Supplementary Data 1

Description: Proteins identified by liquid chromatography–tandem mass spectrometry of anti-GFP immunoprecipitations.

File name: Supplementary Data 2

Description: Identification of putative light-dependent NPH3-interacting proteins.
