# Supplementary material for: Regulation of plant phototropic growth by NPH3/RPT2-like substrate phosphorylation and 14-3-3 binding
Source: Nat Commun. 2021 Oct 21;12:6129. doi: 10.1038/s41467-021-26333-5 (PMC8531357; doi:10.1038/s41467-021-26333-5)
Supplement: Supplementary file 6 — Reporting Summary [file 41467_2021_26333_MOESM6_ESM.pdf]

Corresponding author(s): Stuart Sullivan
John M. Christie

Last updated by author(s): Sep 13, 2021

Reporting Summary

Nature Portfolio wishes to improve the reproducibility of the work that we publish. This form provides structure for consistency and transparency in reporting. For further information on Nature Portfolio policies, see our Editorial Policies and the Editorial Policy Checklist.

Statistics

For all statistical analyses, confirm that the following items are present in the figure legend, table legend, main text, or Methods section.

n/a	Confirmed	
☐	☒	The exact sample size (*n*) for each experimental group/condition, given as a discrete number and unit of measurement
☐	☒	A statement on whether measurements were taken from distinct samples or whether the same sample was measured repeatedly
☐	☒	The statistical test(s) used AND whether they are one- or two-sided<br>*Only common tests should be described solely by name; describe more complex techniques in the Methods section.*
☒	☐	A description of all covariates tested
☒	☐	A description of any assumptions or corrections, such as tests of normality and adjustment for multiple comparisons
☐	☒	A full description of the statistical parameters including central tendency (e.g. means) or other basic estimates (e.g. regression coefficient) AND variation (e.g. standard deviation) or associated estimates of uncertainty (e.g. confidence intervals)
☐	☒	For null hypothesis testing, the test statistic (e.g. *F*, *t*, *r*) with confidence intervals, effect sizes, degrees of freedom and *P* value noted<br>*Give P values as exact values whenever suitable.*
☒	☐	For Bayesian analysis, information on the choice of priors and Markov chain Monte Carlo settings
☒	☐	For hierarchical and complex designs, identification of the appropriate level for tests and full reporting of outcomes
☒	☐	Estimates of effect sizes (e.g. Cohen's *d*, Pearson's *r*), indicating how they were calculated

Our web collection on statistics for biologists contains articles on many of the points above.

Software and code

Policy information about availability of computer code

Data collection	QCapture Pro 7<br>Fiji ImageJ 1.53c
Data analysis	Fiji ImageJ 1.53c<br>GraphPad Prism 9.01<br>Oriana 4.02<br>Microsoft Excel 16.51

For manuscripts utilizing custom algorithms or software that are central to the research but not yet described in published literature, software must be made available to editors and reviewers. We strongly encourage code deposition in a community repository (e.g. GitHub). See the Nature Portfolio guidelines for submitting code & software for further information.

Data

Policy information about availability of data

All manuscripts must include a data availability statement. This statement should provide the following information, where applicable:

- Accession codes, unique identifiers, or web links for publicly available datasets
- A description of any restrictions on data availability
- For clinical datasets or third party data, please ensure that the statement adheres to our policy

The authors declare that all data presented in this study are available in the figures and the accompanying Supplementary Information file. Mass spectrometry proteomics data have been deposited to the ProteomeXchange Consortium via the PRIDE partner repository with the data set identifier PXD028698 [https://

www.ebi.ac.uk/pride/archive/projects/PXD028698]. Data that support the study are available from the corresponding authors upon reasonable request. Source data are provided with this paper.

Field-specific reporting

Please select the one below that is the best fit for your research. If you are not sure, read the appropriate sections before making your selection.

☒ Life sciences ☐ Behavioural & social sciences ☐ Ecological, evolutionary & environmental sciences

For a reference copy of the document with all sections, see nature.com/documents/nr-reporting-summary-flat.pdf

Life sciences study design

All studies must disclose on these points even when the disclosure is negative.

Sample size	Sample sizes were chosen based on past experience and typical sample sizes reported in the literature.
Data exclusions	No data were excluded.
Replication	All attempts of replication were successful. Numbers of replicates for each experiment provided within the manuscript.
Randomization	For petiole positioning plants were randomly positioned and positions regularly changed within the growth chamber.
Blinding	Due to the nature of the experimental setup blinding was not practical for this work as the experimenters analyzed the results.

Reporting for specific materials, systems and methods

We require information from authors about some types of materials, experimental systems and methods used in many studies. Here, indicate whether each material, system or method listed is relevant to your study. If you are not sure if a list item applies to your research, read the appropriate section before selecting a response.

Materials & experimental systems

n/a	Involved in the study	
☐	☒	Antibodies
☒	☐	Eukaryotic cell lines
☒	☐	Palaeontology and archaeology
☒	☐	Animals and other organisms
☒	☐	Human research participants
☒	☐	Clinical data
☒	☐	Dual use research of concern

Methods

n/a	Involved in the study	
☒	☐	ChIP-seq
☒	☐	Flow cytometry
☒	☐	MRI-based neuroimaging

Antibodies

Antibodies used	anti-GST monoclonal antibody (Merck ,05-782), anti-HA monoclonal antibody (clone 3F10, Merck 11867423001), anti-GFP-HFP monoclonal antibody (clone GG4-2C2.12.10, Miltenyi Biotech, 130-091-833), anti-thiophosphoester monoclonal antibody (clone 51-8, Abcam ,ab133473), anti-UGPase antibody (AgriSera, AS05 086), anti-phot1 polyclonal antibodies (Cho et al. Plant Physiol 2007, 143:517-529), anti-NPH3 purified polyclonal antibodies (this study - custom antibodies against peptides IPNRKTLIEATPQSF and GVDHPPPRKPRRWRN, Eurogentec), polyclonal antibodies raised against phosphorylated S744 of NPH3 (this study - custom antibodies against peptide KPRRWRNpSIS, Eurogentec), anti-rabbit HRP (Promega, W4011) anti-mouse HRP (Promega, W4021) anti-rat HRP (Dako, P0450), anti-goat HRP (Promega, V8051)
Validation	anti-GST antibody https://www.sigmaaldrich.com/GB/en/product/mm/05782<br>anti-HA antibody https://www.sigmaaldrich.com/GB/en/product/roche/roahaha?context=product<br>anti-GFP-HFP antibody https://www.miltenyibiotec.com/GB-en/products/gfp-antibody-gg4-2c2-12-10.html#gref<br>anti-thiophosphoester antibody https://www.abcam.com/thiophosphate-ester-antibody-51-8-ab133473.html<br>anti-UGPase antibody https://www.agrisera.com/en/artiklar/ugpase-udp-glucose-pyrophosphorylase-marker-of-cytoplasm.html<br>anti-phot1 antibody Cho et al. Plant Physiol 2007, 143:517-529<br>anti-NPH3 antibody - this study, validated in Figure 4<br>anti-phosphorylated S744 of NPH3 antibody - this study, validated in Figure 4<br>anti-rabbit HRP https://www.promega.co.uk/products/protein-detection/primary-and-secondary-antibodies/anti-rabbit-igg-h-and-l-hrp-conjugate/?catNum=W4011#specifications<br>anti-mouse HRP https://www.promega.co.uk/products/protein-detection/primary-and-secondary-antibodies/anti_mouse-igg-h-and-l-hrp-conjugate/?catNum=W4021<br>anti-goat HRP https://www.promega.co.uk/products/protein-detection/primary-and-secondary-antibodies/donkey-anti-goat-igg-hrp/?catNum=V8051
